# Merlin immunohistochemistry is a reliable surrogate marker for *NF2* gene alterations in meningioma

**DOI:** 10.1111/his.15539

**Published:** 2025-08-15

**Authors:** Mayu Kashiwagi‐Hakozaki, Masako Ikemura, Satoru Miyawaki, Yu Teranishi, Atsushi Okano, Nobuhito Saito, Tetsuo Ushiku

**Affiliations:** ^1^ Department of Pathology, Graduate School of Medicine The University of Tokyo Tokyo Japan; ^2^ Department of Neurosurgery, Faculty of Medicine The University of Tokyo Tokyo Japan; ^3^ Present address: Department of Pathology Tokyo Women's Medical University Tokyo Japan; ^4^ Present address: Department of Pathology Toranomon Hospital Tokyo Japan

**Keywords:** immunohistochemistry, meningioma, Merlin, *NF2*

## Abstract

**Aims:**

Molecular characterization as well as histologic grading has proven useful in predicting the biological behaviour of meningiomas. *NF2* gene alterations, that is 22q loss and/or *NF2* mutation, are the most common genetic driver events in meningioma and are associated with tumour location, histological subtype and aggressiveness. This study aimed to evaluate the utility of immunostaining for Merlin, the *NF2*‐encoded protein, as a surrogate marker for detecting *NF2* gene alterations.

**Methods and results:**

We analysed 109 CNS WHO Grade I meningiomas, comparing the Merlin immunostaining patterns with *NF2* gene status. Complete loss of Merlin expression was observed in 46 of 51 (90%) meningiomas with *NF2* alterations, whereas it was observed in 5 of 58 (9%) meningiomas without *NF2* alterations. Therefore, Merlin immunohistochemistry could achieve relatively high sensitivity (90%, 95% confidence interval [CI]: 79%–97%) and specificity (91%, 95% CI: 81%–97%) in detecting *NF2* alterations. Although some cases required careful interpretation, interobserver agreement was high at 93%.

**Conclusions:**

These findings suggest that Merlin immunohistochemistry could serve as a reliable and practical surrogate marker for *NF2* alterations in meningioma. It may support molecular‐based classification and risk stratification in clinical and research settings. However, caution is warranted in cases with cautery tissue damage and prominent macrophages, which can obscure staining patterns.

## Graphical abstract

This study found that complete loss of Merlin immunostaining could serve as a reliable surrogate marker for *NF2* gene alterations in low‐grade meningioma. We also introduce some pitfalls in the interpretation of its staining pattern.

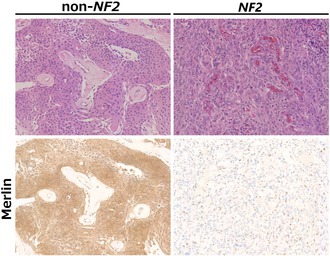

**Abbreviations**
CI: confidence interval
CNS: central nervous system
CNV: copy number variation
IHC: immunohistochemistry
WHO CNS: the WHO Classification of Tumours of the Central Nervous System

## Introduction

Meningioma is the most common primary tumour of the primary central nervous system tumours, accounting for 40.8% of all CNS tumours.^1^ Meningiomas have been graded based on morphological features such as histological subtypes, mitotic activity, brain parenchymal invasion and other minor histological criteria.^2^ However, grading based on morphology alone has been shown to have limited power in predicting tumour aggressiveness and patient prognosis.^3^ The 5th edition of the WHO Classification of Tumours of the Central Nervous System (WHO CNS 5th)^4^ first introduced molecular alterations for defining the grade of meningioma, with *CDKN2A/B* deletion and *TERT* promoter mutations as grade 3 defining parameters.

Recent research has shown that meningiomas are a genetically and epigenetically heterogeneous group of tumours and that molecularly integrated classification is useful for appropriate treatment selection and prognosis prediction.^5^, ^6^, ^7^, ^8^, ^9^ Among them, several driver gene mutations have been identified to be closely associated with anatomical location, histological subtype and tumour behaviour.^10^, ^11^, ^12^, ^13^, ^14^, ^15^ *NF2* is a tumour suppressor gene located on chromosome 22q12.^16^, ^17^ *NF2* gene aberration (loss of chromosome 22q and/or mutations in *NF2*) is the most common driver abnormality in meningiomas, which is identified in 40–60% of sporadic meningiomas.^18^, ^19^ In meningiomas without *NF2* alterations, mutations in *TRAF7, KLF4, AKT1, SMO, PIK3CA* and *POLR2A* are recurrent alterations.^10^, ^12^, ^13^ *NF2* aberrations are known to be enriched in transitional and fibrous meningioma in the intracranial skull base and psammomatous meningioma in the spinal cord.^14^ *NF2* alterations are also associated with an aggressive phenotype of meningiomas, and they were commonly found in high‐grade meningioma.^20^, ^21^, ^22^ In addition, *NF2* alterations have been reported to be linked to a worse prognosis in supratentorial CNS WHO grade I meningiomas.^23^

Copy number variations (CNVs), including losses on 1p, 6p/q, 10q, 14q, 18p/q, and gains on 2p/q, 3p, 4p/q, 7p/q, 8p/q, 13p/q, are documented especially in high‐grade *NF2*‐altered meningioma.^7^, ^24^, ^25^ Recent multiomics analyses also suggested that groups of meningioma with multiple CNVs in addition to *NF2* alterations tended to show poorer outcomes than a group of *NF2*‐altered meningioma without CNVs and a group of *NF2‐*intact meningioma.^6^, ^26^, ^27^ Among them, loss of 1p is most frequently observed^7^, ^25^, ^28^, ^29^ and reported as an independent marker for adverse clinical outcomes,^7^, ^24^, ^30^ so 1p loss is estimated as the first CNV occurring after 22q loss in high‐grade meningioma with *NF2* gene alteration.^31^, ^32^ Based on these findings, cIMPACT‐NOW update 8^33^ proposed that meningioma with 1p deletion combined with 22q loss and/or *NF2* oncogenic variants should be graded at least CNS WHO grade 2, even in histologically otherwise benign tumours. Thus, detection for 22q loss and *NF2* mutations might be required to grade meningioma in the next edition of WHO classification.

*NF2* alterations were usually examined by sequencing analysis, which is often difficult to perform in routine practice. A potential surrogate marker is immunohistochemistry (IHC) of Merlin, a protein encoded by *NF2*. A few studies on schwannoma and mesothelioma have shown that Merlin immunohistochemistry is correlated with *NF2* molecular status.^34^, ^35^, ^36^ Given the limited information about the predictive value of Merlin IHC in detecting *NF2* alterations in meningioma, this study investigated the immunostaining pattern of Merlin in a case series of CNS WHO grade I meningiomas and compared it with their genetic abnormalities. The staining characteristics were evaluated in detail, and pitfalls in their interpretation were presented.

## Materials and methods

### Case Selection

This study was approved by our institutional review board (approval number: G10028). Informed consent was obtained from all patients. WHO grade I meningiomas surgically resected at The University of Tokyo Hospital, Tokyo, Japan, between 2010 and 2018 were retrospectively retrieved from the institutional archive. The pathological diagnosis was reviewed by expert pathologists based on the 2016 WHO classification. Patients with incomplete clinical or genetic information or a history of meningioma treatment were excluded.

### Immunohistochemistry and Interpretation

Immunohistochemical staining was performed on representative sections cut at 3 μm thickness from the formalin‐fixed, paraffin‐embedded block, using a Ventana autostainer (Roche Diagnostics, Basel, Switzerland). Immunostaining for Merlin was performed using a monoclonal antibody (clone D3S3W, 1:50; Cell signalling technology, Danvers, MA, USA) with the following optimized conditions: heat‐induced epitope antigen retrieval for 64 min using CC1 buffer at high pH, primary antibody incubation for 32 min and the OptiView DAB IHC detection kit (Roche Diagnostics). Merlin staining was considered retained when tumour cells showed positive cytoplasmic staining, regardless of intensity. Loss of Merlin immunoreactivity was defined by the complete absence of cytoplasmic staining in the tumour cells, with positive staining in the background non‐neoplastic cells, such as inflammatory cells and vascular endothelial cells, as positive internal controls. The staining pattern was scored independently by two pathologists (MKH and MI) who were unaware of the molecular *NF2* status. The scoring results were then compared, and any discrepancies or difficulties were discussed to reach a consensus. Some selected cases were also immunostained with monoclonal antibodies for CD3 (clone LN10, 1:50; Leica Biosystems, Nussloch, Germany), CD20 (clone L26, 1:1; Roch Diagnostics) and CD68 (clone Kp‐1, 1:200; Agilent technologies, Santa Clara, USA).

### Molecular Data

The molecular data for this series of meningioma cases have been reported previously.^37^ This included microsatellite analysis to detect 22q loss, direct Sanger sequencing of all exons of *NF2* and direct Sanger sequencing for mutational hotspots of *AKT1*, *KLF4*, *SMO*, *POLR2A*. Meningiomas with *NF2* mutation and/or 22q loss were defined as ‘*NF2* meningiomas’, while others were defined as ‘non‐*NF2* meningiomas’.

### Statistical Analysis

To evaluate performance, we assessed the sensitivity, specificity, positive predictive value and negative predictive value for Merlin staining to detect *NF2* alterations. We also estimated the 95% confidence interval (CI) of each value. All statistical analyses were performed using the Easy R (EZR) plug‐in (Saitama Medical Center, Jichi Medical University, Saitama, Japan),^38^ which is a graphical user interface for R (The R Foundation for Statistical Computing, Vienna, Austria).

## Results

### *NF2* Genetic Alterations

A total of 109 cases of CNS WHO grade I meningioma were included in this study (Table 1). The genetic alterations detected in each histological subtype are summarized in Table 2. *NF2* alterations were detected in 51 cases (46.8%) of all meningiomas: 33 cases with both 22q loss and *NF2* mutation, 16 cases with 22q loss only and two cases with *NF2* mutation only. All cases with 22q loss exhibited heterozygous (monoallelic) 22q loss. Specifically, *NF2* meningioma accounts for 80.0% (12/15) of fibrous meningioma, 63.9% (23/36) of transitional meningioma and 27.8% (15/54) of meningothelial meningioma. Mutations in *AKT1*, *KLF4*, *POLR2A* or *SMO* were mostly detected in meningothelial or transitional meningioma, except for three cases: one case of angiomatous meningioma with *KLF4* mutation, one case of secretory meningioma with *KLF4* mutation and one case of psammomatous meningioma with *AKT1* mutation. None of the 15 cases of fibrous meningioma had any of these four mutations.

**Table 1:** Clinicopathologic characteristics of patients

Total	*N* = 109
Sex	
Female	83 (76.1%)
Male	26 (23.9%)
*NF2* alteration	
*NF2*	51 (46.8%)
Non‐*NF2*	58 (53.2%)
Pathological diagnosis	
Meningothelial	54 (49.5%)
Transitional	36 (33.0%)
Fibrous	15 (13.8%)
Angiomatous	2 (1.8%)
Secretory	1 (0.9%)
Psammomatous	1 (0.9%)

**Table 2:** Distribution of genetic alterations in different histological subtypes of meningioma

Histological subtypes	*NF2*			Non‐*NF2*					Total
	22q loss and *NF2* mut	22q loss	*NF2* mut	*AKT1*	*KLF4*	*POLR2A*	*SMO*	Others	
Meningothelial	8	6	1	15	4	5	0	15	54
Transitional	17	6	0	5	1	2	1	4	36
Fibrous	8	3	1	0	0	0	0	3	15
Angiomatous	0	1	0	0	1	0	0	0	2
Secretory	0	0	0	0	1	0	0	0	1
Psammomatous	0	0	0	1	0	0	0	0	1

### Merlin Staining Patterns and *NF2* Status

**Figure 1:**
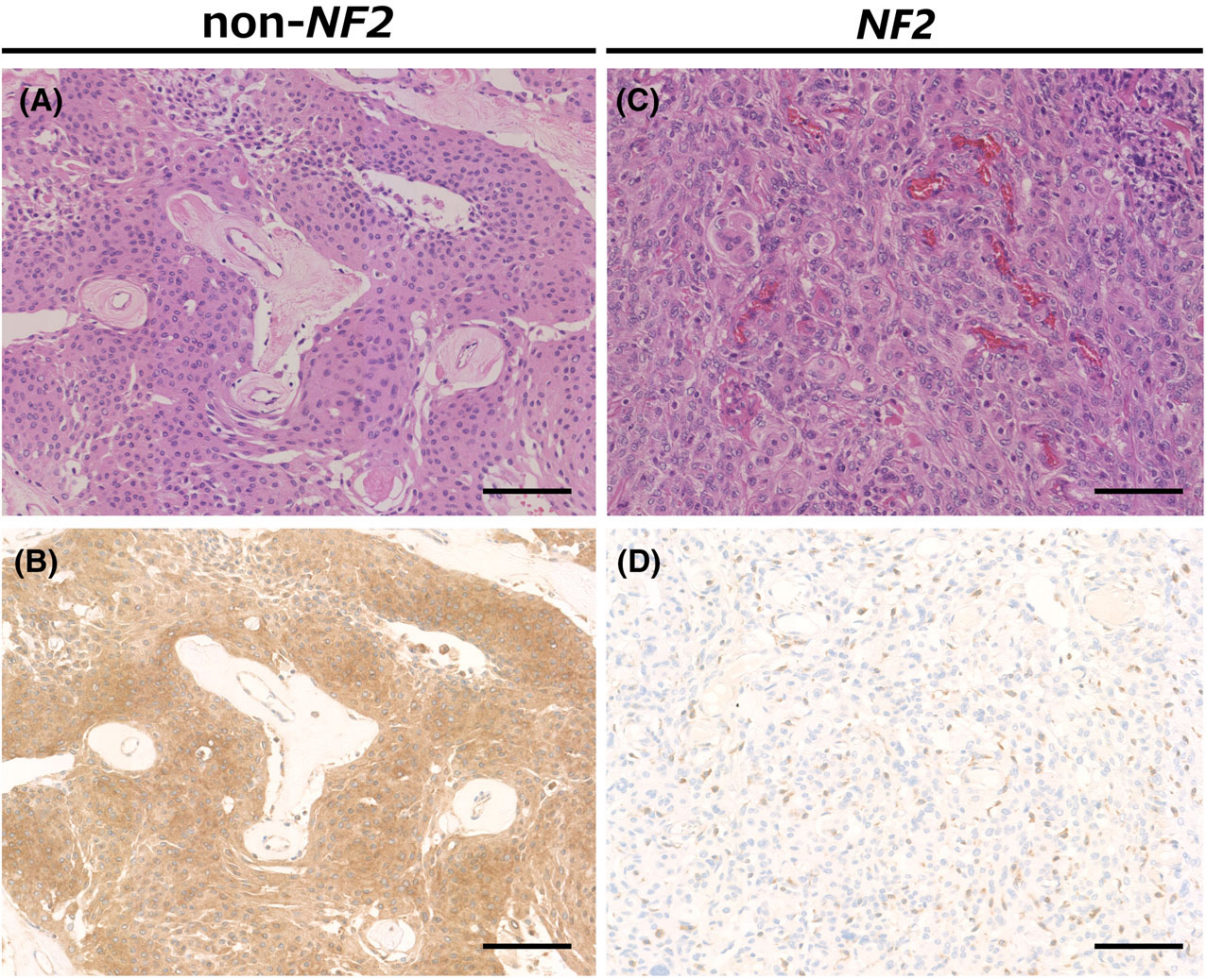
Representative patterns of Merlin immunohistochemistry. (**A**, **B**) Meningothelial meningioma, harbouring *AKT1* mutation (non‐*NF2* meningioma). (**A**) HE stains. (**B**) Diffuse and strong immunoreactivity for Merlin in tumour cytoplasm. (**C**, **D**) Meningothelial meningioma, harbouring 22q loss and *NF2* mutation (*NF2* meningioma). (**C**) HE stains. (**D**) Tumour cells are completely negative for Merlin, whereas non‐neoplastic inflammatory cells show Merlin expression serving as an internal positive control (Scale bar: 100 μm).

*NF2* molecular status and Merlin immunostaining pattern of all cases are shown in Table 3. Loss of Merlin immunoreactivity was detected in 46.8% (51/109) of all meningioma cases. The loss of staining was uniformly observed in the evaluable tumour areas. Representative cases of Merlin‐lost *NF2* meningiomas and Merlin‐retained non‐*NF2* meningiomas are shown in Figure 1. Of the 51 *NF2* meningiomas, 46 showed loss of Merlin staining, whereas 53 of 58 non‐*NF2* meningiomas retained Merlin staining (sensitivity: 90.2% [95% CI: 78.6%–96.7%], specificity: 91.4% [95% CI: 81.0%–97.1%], positive predictive value: 90.2% [95% CI: 78.6%–96.7%], negative predictive value: 91.4% [95% CI: 81.0%–97.1%]).

**Table 3:** Immunohistochemical staining patterns of Merlin and *NF2* molecular alteration

	Immunohistochemical pattern of Merlin			
	Lost	Retained	Total	
*NF2*	46	5	51	Sensitivity = 90.2%
Non‐*NF2*	5	53	58	Specificity = 91.4%
*NF2*				Sensitivity
22q loss + *NF2* mutation	33	0	33	100%
22q loss only	11	5	16	68.8%
*NF2* mutation only	2	0	2	100%
Non‐*NF2*				Specificity
*AKT1*	0	21	21	100%
*KLF4*	0	7	7	100%
*POLR2A*	0	7	7	100%
*SMO*	0	1	1	100%
Others	5	17	22	77.3%

Some cases showed discrepancies between the pattern of Merlin immunostaining and the presence of *NF2* gene alteration. Five cases of non‐*NF2* meningioma showed loss of Merlin staining, including three cases of fibrous meningioma, one case of transitional meningioma and one case of meningothelial meningioma. In all five cases, no hotspot mutations in *AKT1*, *KLF4*, *SMO* and *POLR2A* were detected. On the contrary, there were five cases of *NF2* meningioma with retained IHC pattern of Merlin. All these cases were meningothelial meningioma. All *NF2* meningioma with retained Merlin IHC had only 22q loss, whereas no *NF2* mutations were detected.

### Interobserver Agreement for Merlin Immunohistochemistry

**Figure 2:**
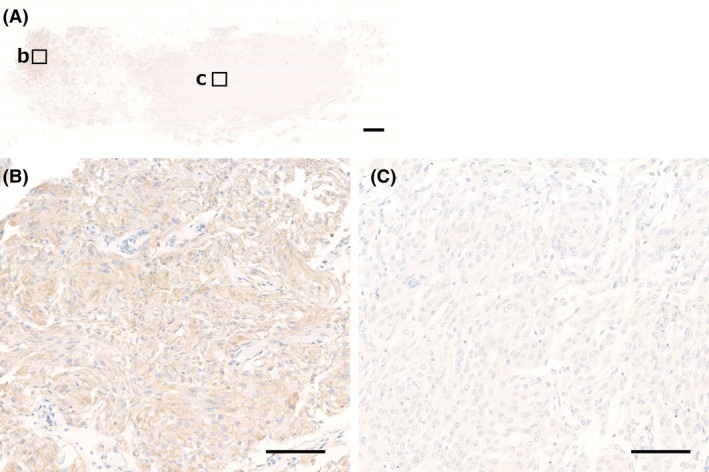
Challenges in the interpretation of Merlin immunostaining, Merlin‐retained pattern. (**A–C**) Meningothelial meningioma, harbouring *AKT1* mutation (non‐*NF2* meningioma). Staining of Merlin. (**B**) Tumour cells show block‐positive for Merlin in the peripheral zones of the specimen. (**C**) Both tumour cells and inflammatory cells (positive internal controls) are negative for Merlin in the central zone of the specimen, suggestive of staining artefacts (Scale bar: (**A**) 5 mm, (**B**, **C**) 100 μm).

**Figure 3:**
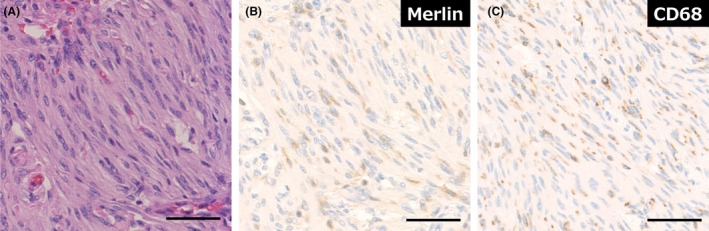
Challenges in the interpretation of Merlin immunostaining, Merlin‐lost pattern. (**A**) Transitional meningioma, harbouring 22q loss and *NF2* mutation (*NF2* meningioma) with marked inflammatory cell infiltration. HE stains. (**B**, **C**) Immunostaining of Merlin (**B**) and CD68 (**C**). Abundant CD68‐positive macrophages, showing the retained pattern of Merlin, exist in the tumour. Tumour cells show no Merlin reactivity (Scale bar: 50 μm).

The two observers were in agreement in 97 (89%) cases, whereas 12 (11%) cases were difficult to interpret Merlin IHC and required discussion to reach a consensus. In 6 of the 58 Merlin‐preserved cases, it was difficult to assess the staining pattern because they showed only partial and weak Merlin staining. The staining intensity within a slide was often heterogeneous, especially in large specimens. In such cases, false Merlin‐negative areas without staining of the internal positive control were detected in the sample, suggesting staining artefacts (Figure 2). In 6 of the 51 cases of Merlin loss, careful evaluation is required to distinguish between the admixture of non‐neoplastic cells and tumour cells. In three cases, a prominent infiltration of Merlin‐positive inflammatory cells made scoring difficult (Figure 3). IHC analysis revealed numerous CD68‐positive round or spindle‐shaped macrophages infiltrating the tumour, while the tumour cells exhibited no immunopositivity for Merlin. Abundant macrophage infiltration was relatively common in fibrous meningioma and requires careful interpretation. In addition, in the other three Merlin‐lost cases, non‐neoplastic brain tissue or meningeal cells showing block‐positive for Merlin were seen within the tumour, making scoring confusing.

## Discussions

*NF2* alteration has been recognized as an important factor for molecular subgrouping of meningiomas, and it is potentially a useful diagnostic and prognostic marker. In this study, we showed that loss of Merlin immunostaining provided a high concordance with *NF2* alteration in Grade I meningioma (sensitivity: 90.2%, specificity: 91.4%, positive predictive value: 90.2%, negative predictive value: 91.4%). Therefore, Merlin IHC may serve as a useful surrogate marker for the molecular status of *NF2*, instead of costly and time‐consuming genomic analysis.

A recent study on pleural mesothelioma^35^ showed that Merlin IHC was concordant with *NF2* molecular status in 77% of tumours (65/84), using monoclonal antibody clone D1D8, that recognizes an epitope near the N‐terminus of Merlin. Another study^38^ of mesothelioma reported that Merlin antibody clone D3S3W, which binds an epitope near the C‐terminus, showed a more diffuse and strong staining pattern of Merlin. This observation suggests that clone D3S3W is suitable as a surrogate marker for *NF2* gene alteration. Our study used the latter clone D3S3W for Merlin IHC and showed a high concordance rate (90.8%) between Merlin IHC and *NF2* gene status.

In a small subset of cases, there was a discrepancy between the *NF2* status and the Merlin IHC results. On the one hand, there were five cases of non‐*NF2* meningioma displaying a loss of Merlin IHC pattern. In all five cases, mutations in *AKT1*, *KLF4*, *SMO1*, *POLR2A* were also not detected. In such cases, epigenetic changes in *NF2* may lead to loss of Merlin expression. One study reported that methylation of three CpG sites in the *NF2* promoter region was found in up to 60% of schwannomas and correlated with decreased mRNA expression.^39^ Another study of 88 cases of sporadic meningiomas found that 49% of cases had 22q loss, 24% had somatic *NF2* mutations and 26% had aberrant *NF2* promoter methylation, and that epigenetic *NF2* inactivation was the sole cause of *NF2* deficiency in 17% of meningiomas.^40^ Methylation analysis and investigation of other gene alterations associated with non‐*NF2* meningiomas may be helpful to determine the *NF2* status of these cases. Another possibility is that Merlin was inactivated by μ‐calpain‐mediated proteolysis. Activation of the μ‐calpain system, which mediates proteolytic cleavage and degeneration of Merlin, has been reported to result in loss of Merlin expression in some cases of schwannomas and meningiomas without *NF2* alteration.^41^ However, the role of μ‐calpain system in meningioma tumorigenesis is still controversial.^42^

On the other hand, five cases of *NF2* meningioma showed retained Merlin staining, although the staining intensity was weak in all cases. All these cases had only 22q loss and no *NF2* mutation was detected, suggesting that they might have *NF2* alteration in only a single allele. The heterogeneity of the tumour may have influenced the results, as genetic and immunohistochemical analyses were performed on different parts within each tumour. Further investigation may be needed to elucidate the cause of these discrepancies.

Of note, there were difficulties in the interpretation of Merlin IHC in some cases. Meningioma tissue is usually resected in fragments, and it should be noted that uniform staining is unlikely to be seen because the tumour tissue has undergone surgical artificial damage that affects its staining properties. In addition, the positive staining of non‐neoplastic cells, such as macrophages, may be predominant and obscure the Merlin‐deficient tumour cells. Especially in fibrous meningioma, it is often difficult to distinguish markedly infiltrated spindle‐shaped macrophages from tumour cells. For such cases, detecting macrophages with CD68 immunostaining is useful for interpretation. It is necessary to carefully compare the staining patterns of the inner positive control cells and tumour cells.

In summary, Merlin immunohistochemistry can be a reliable surrogate marker for *NF2* alteration in meningioma in daily pathological practice, although there are some pitfalls to properly interpret its staining pattern. Merlin immunohistochemistry, if combined with the evaluation of 1p loss using FISH, might contribute to effective and easy prognosis prediction of meningioma.

## Author contributions

MK‐H and MI designed the study and performed histological examination. SM, YT, AO and NS provided clinical data and performed molecular analysis. MK‐H drafted the manuscript, and other authors revised it critically. TU supervised the project. All authors read and approved the final manuscript.

## Conflict of interest statement

The authors declare that they have no conflict of interest.

## Funding information

The authors declare that they have no funding.

## Consent to participate

Informed consent was obtained from all patients.

## Consent for publication

Obtained.

## Data Availability

The data that support the findings of this study are available from the corresponding author upon reasonable request.

## References

[1] Ostrom QT , Price M , Neff C *et al*. CBTRUS statistical report: primary brain and other central nervous system tumors diagnosed in the United States in 2016‐2020. Neuro‐Oncology 2023; 25; iv1–iv99. 37793125 10.1093/neuonc/noad149 PMC10550277

[2] Louis DN , Ohgaki H , Wiestler OD *et al*. The 2007 WHO classification of tumours of the central nervous system. Acta Neuropathol. 2007; 114; 97–109. 17618441 10.1007/s00401-007-0243-4 PMC1929165

[3] Mirian C , Duun‐Henriksen AK , Juratli T *et al*. Poor prognosis associated with TERT gene alterations in meningioma is independent of the WHO classification: an individual patient data meta‐analysis. J. Neurol. Neurosurg. Psychiatry 2020; 91; 378–387. 32041819 10.1136/jnnp-2019-322257

[4] Louis DN , Perry A , Wesseling P *et al*. The 2021 WHO classification of tumors of the central nervous system: a summary. Neuro‐Oncology 2021; 23; 1231–1251. 34185076 10.1093/neuonc/noab106 PMC8328013

[5] Sahm F , Schrimpf D , Stichel D *et al*. DNA methylation‐based classification and grading system for meningioma: a multicentre, retrospective analysis. Lancet Oncol. 2017; 18; 682–694. 28314689 10.1016/S1470-2045(17)30155-9

[6] Nassiri F , Liu J , Patil V , Mamatjan Y *et al*. A clinically applicable integrative molecular classification of meningiomas. Nature 2021; 597; 119–125. 34433969 10.1038/s41586-021-03850-3 PMC11604310

[7] Maas SLN , Stichel D , Hielscher T *et al*. Integrated molecular‐morphologic meningioma classification: a multicenter retrospective analysis, retrospectively and prospectively validated. J. Clin. Oncol. 2021; 39; 3839–3852. 34618539 10.1200/JCO.21.00784 PMC8713596

[8] Berghoff AS , Hielscher T , Ricken G *et al*. Prognostic impact of genetic alterations and methylation classes in meningioma. Brain Pathol. 2022; 32; e12970. 35213082 10.1111/bpa.12970 PMC8877750

[9] Franca RA , Della Monica R , Corvino S *et al*. WHO grade and pathological markers of meningiomas: clinical and prognostic role. Pathol. Res. Pract. 2023; 243; 154340. 36738518 10.1016/j.prp.2023.154340

[10] Clark VE , Erson‐Omay EZ , Serin A *et al*. Genomic analysis of non‐NF2 meningiomas reveals mutations in TRAF7, KLF4, AKT1, and SMO. Science 2013; 339; 1077–1080. 23348505 10.1126/science.1233009 PMC4808587

[11] Brastianos PK , Horowitz PM , Santagata S *et al*. Genomic sequencing of meningiomas identifies oncogenic SMO and AKT1 mutations. Nat. Genet. 2013; 45; 285–289. 23334667 10.1038/ng.2526 PMC3739288

[12] Clark VE , Harmancı AS , Bai H *et al*. Recurrent somatic mutations in POLR2A define a distinct subset of meningiomas. Nat. Genet. 2016; 48; 1253–1259. 27548314 10.1038/ng.3651 PMC5114141

[13] Abedalthagafi M , Bi WL , Aizer A *et al*. Oncogenic PI3K mutations are as common as AKT1 and SMO mutations in meningioma. Neuro‐Oncology 2016; 18; 649–655. 26826201 10.1093/neuonc/nov316 PMC4827048

[14] Youngblood MW , Duran D , Montejo JD *et al*. Correlations between genomic subgroup and clinical features in a cohort of more than 3000 meningiomas. J. Neurosurg. 2019; 133; 1345–1354. 31653806 10.3171/2019.8.JNS191266

[15] Yuzawa S , Nishihara H , Tanaka S . Genetic landscape of meningioma. Brain Tumor Pathol. 2016; 33; 237–247. 27624470 10.1007/s10014-016-0271-7

[16] Trofatter JA , MacCollin MM , Rutter J *et al*. A novel moesin‐, ezrin‐, radixin‐like gene is a candidate for the neurofibromatosis 2 tumor suppressor. Cell 1993; 72; 791–800. 8453669 10.1016/0092-8674(93)90406-g

[17] Rouleau GA , Merel P , Lutchman M *et al*. Alteration in a new gene encoding a putative membrane‐organizing protein causes neuro‐fibromatosis type 2. Nature 1993; 363; 515–521. 8379998 10.1038/363515a0

[18] Ruttledge MH , Sarrazin J , Rangaratnam S *et al*. Evidence for the complete inactivation of the NF2 gene in the majority of sporadic meningiomas. Nat. Genet. 1994; 6; 180–184. 8162072 10.1038/ng0294-180

[19] De Vitis LR , Tedde A , Vitelli F *et al*. Screening for mutations in the neurofibromatosis type 2 (NF2) gene in sporadic meningiomas. Hum. Genet. 1996; 97; 632–637. 8655144 10.1007/BF02281874

[20] Bi WL , Greenwald NF , Abedalthagafi M *et al*. Genomic landscape of high‐grade meningiomas. NPJ Genom. Med. 2017; 2; 15. 28713588 10.1038/s41525-017-0014-7 PMC5506858

[21] Williams EA , Santagata S , Wakimoto H *et al*. Distinct genomic subclasses of high‐grade/progressive meningiomas: NF2‐associated, NF2‐exclusive, and NF2‐agnostic. Acta Neuropathol. Commun. 2020; 8; 171. 33087175 10.1186/s40478-020-01040-2 PMC7580027

[22] Riemenschneider MJ , Perry A , Reifenberger G . Histological classification and molecular genetics of meningiomas. Lancet Neurol. 2006; 5; 1045–1054. 17110285 10.1016/S1474-4422(06)70625-1

[23] Teranishi Y , Okano A , Miyawaki S *et al*. Clinical significance of NF2 alteration in grade I meningiomas revisited; prognostic impact integrated with extent of resection, tumour location, and Ki‐67 index. Acta Neuropathol. Commun. 2022; 10; 76. 35570314 10.1186/s40478-022-01377-w PMC9107722

[24] Driver J , Hoffman SE , Tavakol S *et al*. A molecularly integrated grade for meningioma. Neuro‐Oncology 2022; 24; 796–808. 34508644 10.1093/neuonc/noab213 PMC9071299

[25] Harmancı AS , Youngblood MW , Clark VE *et al*. Integrated genomic analyses of de novo pathways underlying atypical meningiomas. Nat. Commun. 2017; 8; 14433. 28195122 10.1038/ncomms14433 PMC5316884

[26] Bayley JC 5th , Hadley CC , Harmanci AO *et al*. Multiple approaches converge on three biological subtypes of meningioma and extract new insights from published studies. Sci. Adv. 2022; 8; eabm6247. 35108039 10.1126/sciadv.abm6247 PMC11313601

[27] Choudhury A , Magill ST , Eaton CD *et al*. Meningioma DNA methylation groups identify biological drivers and therapeutic vulnerabilities. Nat. Genet. 2022; 54; 649–659. 35534562 10.1038/s41588-022-01061-8 PMC9374001

[28] Maillo A , Orfao A , Espinosa A *et al*. Early recurrences in histologically benign/grade I meningiomas are associated with large tumors and coexistence of monosomy 14 and del(1p36) in the ancestral tumor cell clone. Neuro‐Oncology 2007; 9; 438–446. 17704362 10.1215/15228517-2007-026 PMC1994101

[29] Domingues PH , Sousa P , Otero Á *et al*. Proposal for a new risk stratification classification for meningioma based on patient age, WHO tumor grade, size, localization, and karyotype. Neuro‐Oncology 2014; 16; 735–747. 24536048 10.1093/neuonc/not325 PMC3984558

[30] Maas SLN , Sievers P , Weber DC *et al*. Independent prognostic impact of DNA methylation class and chromosome 1p loss in WHO grade 2 and 3 meningioma undergoing adjuvant high‐dose radiotherapy: comprehensive molecular analysis of EORTC 22042‐26042. Acta Neuropathol. 2023; 146; 837–840. 37855895 10.1007/s00401-023-02642-5 PMC10627973

[31] Ketter R , Urbschat S , Henn W *et al*. Application of oncogenetic trees mixtures as a biostatistical model of the clonal cytogenetic evolution of meningiomas. Int. J. Cancer 2007; 121; 1473–1480. 17557299 10.1002/ijc.22855

[32] Magill ST , Vasudevan HN , Seo K *et al*. Multiplatform genomic profiling and magnetic resonance imaging identify mechanisms underlying intratumor heterogeneity in meningioma. Nat. Commun. 2020; 11; 4803. 32968068 10.1038/s41467-020-18582-7 PMC7511976

[33] Sahm F , Aldape KD , Brastianos P *et al*. cIMPACT‐NOW update 8: clarifications on molecular risk parameters and recommendations for WHO grading of meningiomas. Neuro‐Oncology 2024; 27; noae170. 10.1093/neuonc/noae170 PMC11812049 39212325

[34] Chapel DB , Hornick JL , Barlow J *et al*. Clinical and molecular validation of BAP1, MTAP, P53, and Merlin immunohistochemistry in diagnosis of pleural mesothelioma. Mod. Pathol. 2022; 35; 1383–1397. 35459788 10.1038/s41379-022-01081-z PMC9529776

[35] Martin SD , Cheung S , Churg A . Immunohistochemical demonstration of Merlin/NF2 loss in mesothelioma. Mod. Pathol. 2023; 36; 100036. 36788071 10.1016/j.modpat.2022.100036

[36] Sainz J , Huynh DP , Figueroa K *et al*. Mutations of the neurofibromatosis type 2 gene and lack of the gene product in vestibular schwannomas. Hum. Mol. Genet. 1994; 3; 885–891. 7951231 10.1093/hmg/3.6.885

[37] Okano A , Miyawaki S , Hongo H *et al*. Associations of pathological diagnosis and genetic abnormalities in meningiomas with the embryological origins of the meninges. Sci. Rep. 2021; 11; 6987. 33772057 10.1038/s41598-021-86298-9 PMC7998008

[38] Kanda Y . Investigation of the freely available easy‐to‐use software “EZR” for medical statistics. Bone Marrow Transplant. 2013; 48; 452–458. 23208313 10.1038/bmt.2012.244 PMC3590441

[39] Kino T , Takeshima H , Nakao M *et al*. Identification of the cis‐acting region in the NF2 gene promoter as a potential target for mutation and methylation‐dependent silencing in schwannoma. Genes Cells 2001; 6; 441–454. 11380622 10.1046/j.1365-2443.2001.00432.x

[40] Lomas J , Bello MJ , Arjona D *et al*. Genetic and epigenetic alteration of the NF2 gene in sporadic meningiomas. Genes Chromosomes Cancer 2005; 42; 314–319. 15609345 10.1002/gcc.20141

[41] Kimura Y , Koga H , Araki N *et al*. The involvement of calpain‐independent proteolysis of the tumor suppressor NF2 (merlin) in schwannomas and meningiomas. Nat. Med. 1998; 4; 915–922. 9701243 10.1038/nm0898-915

[42] Ueki K , Wen‐Bin C , Narita Y *et al*. Tight association of loss of Merlin expression with loss of heterozygosity at chromosome 22q in sporadic meningiomas1. Cancer Res. 1999; 59; 5995–5998. 10606247

